# Network pharmacology and molecular dynamics simulations reveal shared mechanisms and myopia-specific targets of atropine in myopia and dry eye disease

**DOI:** 10.1186/s40662-026-00493-1

**Published:** 2026-06-22

**Authors:** Meng Lin, Wanyi Shu, Guo Hua, Zhikang Duan, Jia Qu, Liang Hu

**Affiliations:** 1 National Clinical Research Center for Ocular Diseases, Eye Hospital, Wenzhou Medical University, Wenzhou, 325027 Zhejiang China; 2 School of Ophthalmology and Optometry, Wenzhou Medical University, Wenzhou, 325035 Zhejiang China

**Keywords:** Atropine, Myopia, Dry eye disease, Molecular docking, Molecular dynamics simulation

## Abstract

**Background:**

Atropine is an effective agent for myopia control; however, clinical reports have indicated potential side effects associated with inducing or exacerbating dry eye disease (DED). It remains unclear whether the therapeutic targets involved in atropine-mediated myopia control overlap with those potentially associated with ocular surface effects. Therefore, this study aimed to characterise the shared or distinct target landscape and plausible molecular pathways through which atropine may contribute to myopia control while also being associated with dry eye-related symptoms.

**Methods:**

In silico analysis was performed to explore the molecular interactions of atropine in controlling myopia and DED. The target lists for atropine, myopia, and DED were sourced from six public databases. Gene Ontology (GO) and Kyoto Encyclopedia of Genes and Genomes (KEGG) enrichment analyses identified common and unique pathways. A protein–protein interaction (PPI) network was created and analysed using Cytoscape to identify hub genes using six ranking algorithms. Targets were stratified into shared Class I and myopia-prioritised Class II categories by integrating retinal and pan-ocular expression data. Molecular docking and molecular dynamics (MD) simulations were conducted to assess the binding affinities of atropine to prioritised targets.

**Results:**

A total of 57 shared Class I and 19 Class II myopia-prioritised targets were identified; 19 hub genes were consistently ranked across the six algorithms. Class I targets converge on inflammatory and extracellular-matrix-remodelling pathways. Evidence of ocular expression supports the biological relevance of the prioritised targets in both classes. Docking showed favourable atropine binding to representative Class I targets (EGFR, MMP2, MMP9, and MAPK1) and Class II candidates (PIK3R1 and AKR1B1). MD simulations supported stable complexes for eight atropine-target pairs, with MM-PBSA binding free energy estimates supporting the computational prioritisation.

**Conclusions:**

Shared Class I targets may potentially link the effects of atropine on both myopia control and DED, along with Class II targets specific to myopia, thereby generating testable hypotheses regarding the dual ocular effects of atropine and informing future mechanistic and translational studies.

**Supplementary Information:**

The online version contains supplementary material available at 10.1186/s40662-026-00493-1.

## Background

Atropine, a non-selective muscarinic receptor antagonist, is widely used to manage myopia [1, 2]; low doses of atropine effectively slow axial elongation and cause minimal reversible side effects [1, 3]. Recent studies suggest that topical atropine use may be associated with dry-eye-related symptoms and possible worsening of dry eye disease (DED) [4, 5]. This highlights a paradox regarding how the same drug can benefit the posterior segment by inhibiting myopia while affecting the anterior segment in ways associated with DED. The exact molecular mechanisms underlying this duality remain unclear and require further investigation.

Atropine effectively controls myopia by slowing its progression through various mechanisms. As a non-selective muscarinic antagonist, it affects eye tissues, such as the choroid and sclera, reducing axial elongation [3] and improving microcirculation [6]. In addition, atropine reduces scleral hypoxia, regulates extracellular matrix (ECM) remodelling, and decreases vasoactive intestinal peptide (VIP) expression [7]. Although its effects on ocular tissues and signalling pathways make it a promising treatment, research on how atropine may be associated with dry-eye-related effects remains limited. One study suggested that atropine may contribute to ocular surface injury by causing corneal cell apoptosis and disrupting signalling via reduced *miR-30c-1*, increased SOCS3, and inhibited JAK2/STAT3 pathways, extending beyond its role as a cholinergic antagonist [8]. Experimental evidence has also linked atropine to dopaminergic signalling, scleral ECM remodelling via MMP-mediated mechanisms [3], and ocular surface inflammatory responses [9]. Recent in silico studies have expanded target prioritization workflows by integrating molecular docking, molecular dynamics (MD) simulations, and free-energy-based analyses, providing a useful methodological context for the present study [10–16]. Understanding whether therapeutic targets overlap between myopia and DED has significant biological implications. Target convergence may indicate shared inflammatory, neuromodulatory, and ECM-remodelling pathways involving PI3K-Akt, MAPK, and cytokine signalling cascades [3], whereas distinct targets may point to separable pharmacological axes. Therefore, we stratified candidates into Class I (shared) and Class II (myopia-specific) categories to test whether the dual effects of atropine reflect pleiotropic mechanisms or context-specific targets amenable to selective modulation.

To address this gap, we used an integrated in silico framework to examine the potential target landscape of atropine in myopia and DED. Specifically, this study identified atropine-related targets and disease-associated genes, defined shared and myopia-prioritized target classes, characterized their functional and network properties, incorporated retinal and pan-ocular transcriptomic evidence for biological prioritization, and evaluated representative ligand–target interactions using molecular docking and MD simulations. By distinguishing shared and myopia-prioritized targets, this study sought to clarify whether the dual ocular effects of atropine can be better explained by overlapping stress-response programmes, more selective retinal signalling mechanisms, or a combination of both.

## Methods

### Study design

**Fig. 1:**
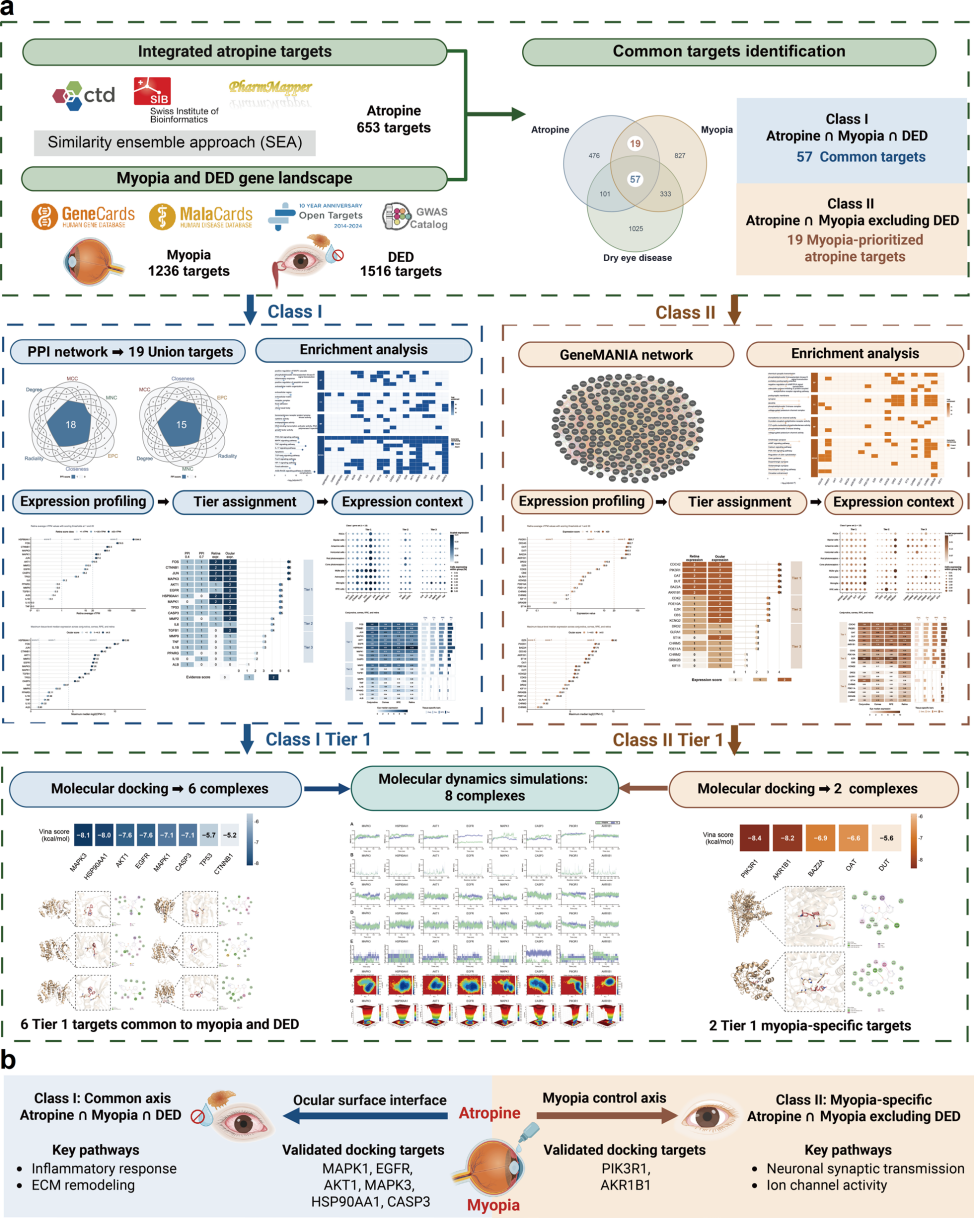
Overview of the study design and key findings. **a** Overall workflow of target identification, class-specific prioritisation, Molecular docking, and MD simulations. **b** Summary of the two main biological axes emerging from the study. MD, molecular dynamics; DED, dry eye disease; ECM, extracellular matrix; PPI, protein–protein interaction

Figure 1 summarizes the workflow of this study. The study first collected candidate targets for atropine and disease-associated genes for myopia and DED from public databases. Overlap analysis was then used to define shared targets (Class I) and myopia-prioritized atropine targets (Class II). These target sets were subsequently characterized using Gene Ontology (GO), Kyoto Encyclopedia of Genes and Genomes (KEGG) enrichment, and protein–protein interaction (PPI) network analyses. To improve the ocular biological relevance, retinal and pan-ocular bulk transcriptomic data, together with retinal single-cell expression patterns, were integrated for target prioritisation. Finally, representative high-priority targets were evaluated using molecular docking and MD simulations **(**Fig. 1a**)**. Figure 1b shows a schematic summary of the two main biological axes identified in this study.

### Drug targets of atropine

The molecular structure and canonical Simplified Molecular Input Line Entry System (SMILES) of atropine were retrieved from PubChem (https://pubchem.ncbi.nlm.nih.gov/, accessed 15 August 2025) [17]. Candidate atropine-related targets were then identified from four online databases, namely SwissTargetPrediction (http://www.swisstargetprediction.ch/, accessed 15 August 2025) [18], Similarity Ensemble Approach (SEA, https://sea.bkslab.org/, accessed 15 August 2025) [19], the Comparative Toxicogenomics Database (CTD, http://ctdbase.org/, accessed 15 August 2025) [20], and PharmMapper (http://www.lilab-ecust.cn/pharmmapper, accessed 15 August 2025) [21], with retrieval restricted to *Homo sapiens*. Targets from the four resources were merged and standardised to official human gene symbols using UniProt (https://www.uniprot.org/; accessed 15 August 2025) [22]. The resulting atropine-target interaction network was visualised using Cytoscape 3.9.1 (https://cytoscape.org/, accessed 15 August 2025). Additional database-specific retrieval details are provided in the Additional file 1.

### Disease-associated targets for myopia and DED

Disease-associated genes for myopia and DED were compiled from GeneCards (https://www.genecards.org/, accessed 16 August 2025) [23], MalaCards (https://www.malacards.org/, accessed 16 August 2025) [24], the Open Targets Platform (https://platform.opentargets.org/, accessed 16 August 2025) [25], and the NHGRI-EBI Genome-wide association study (GWAS) Catalogue (https://www.ebi.ac.uk/gwas/). Non-GWAS resources were restricted to *Homo sapiens*. GWAS-supported disease genes were obtained from the summary statistics accessions GCST90475880 and GCST90475895 for myopia and DED, respectively. Genome-wide significance variants (*P* < 5 × 10^−8^) were pruned into approximately independent association signals using linkage disequilibrium (LD) clumping implemented in PLINK version 1.9 [26], with 1000 Genomes Project reference genotypes matched to the harmonized summary-statistics build [27]. The nearest protein-coding gene to the index variant was designated as the nearest-to-hit gene [28], yielding GWAS-supported candidate genes for myopia and DED. To improve confidence and reduce database-specific noise, only genes identified in at least two non-GWAS resources or supported by at least one database and GWAS (nearest-to-hit), were retained as the final disease-associated targets for subsequent network construction and functional enrichment analyses. Additional retrieval and filtering details are provided in the Additional file 1.

### GO and KEGG enrichment analysis

Targets related to atropine, myopia, and DED were analyzed using the Database for Annotation, Visualization, and Integrated Discovery (DAVID; https://davidbioinformatics.nih.gov, accessed on 16 August 2025) [29] for GO function [30] and KEGG pathway enrichment, focusing on *Homo sapiens*. DAVID was used to explore the gene lists and extract biological insights. Data on biological processes (BP), cellular components (CC), molecular functions (MF), and KEGG were visualized, with the top related terms for each category selected based on false discovery rate-adjusted *P* values.

### PPI network construction

To investigate the interaction architecture of shared atropine-, myopia-, and DED-related targets, we constructed a PPI network using the STRING database (http://string-db.org, accessed on 16 August 2025) [31], restricting the species to *Homo sapiens*. PPI networks were generated under two different confidence thresholds (combined score ≥ 0.40 and ≥ 0.70) to capture both broader and higher-confidence interaction patterns, and the union of interactions passing either threshold was used for downstream analyses. The resulting network was visualized using Cytoscape 3.9.1 (https://cytoscape.org/, accessed 16 August 2025) to identify key hub proteins. Furthermore, to ascertain the top 20 hub targets with the most substantial influence within the network, the CytoHubba plugin in Cytoscape was employed using six distinct ranking algorithms: Maximal Clique Centrality (MCC), Degree, Maximum Neighbourhood Component (MNC), Edge Percolated Component (EPC), Radiality, and Closeness [32]. A Venn diagram was constructed to display the overlapping hub genes identified using six algorithms.

### Integration of retinal and ocular expression evidence and class-specific target prioritization

Transcriptomic support for candidate targets was evaluated using both retina-specific and pan-ocular expression datasets, and targets were prioritised using class-specific empirically defined scoring schemes.

#### Retina expression

Retinal expression data were obtained from the Human Protein Atlas (HPA), which provides consensus RNA-sequencing expression values derived from HPA and Genotype-Tissue Expression (GTEx) datasets (https://www.proteinatlas.org/, accessed on 15 August 2025). For each gene, the consensus normalized transcripts per million (nTPM) value in human retina was extracted, and genes were assigned to three retinal expression score classes using cut-offs of 1 and 20 nTPM (< 1, 1 to < 20, and ≥ 20 nTPM). A lower threshold of 1 nTPM was used in accordance with the HPA convention for detectable transcript expression, whereas 20 nTPM was selected to indicate relatively high retinal expression based on the empirical distribution of retinal nTPM values.

#### Ocular expression

Broader ocular expression was assessed using the Eye in a Disk (EiaD) bulk RNA-sequencing resource implemented in eyeIntegration (https://eyeintegration.nei.nih.gov/, accessed on 10 March 2026) [33], which compiles uniformly processed RNA-sequencing data from multiple human eye tissues. In this study, four anterior and posterior segment tissues directly relevant to myopia and dry eye pathophysiology were considered: the conjunctiva, cornea, retina, and retinal pigment epithelium (RPE). For each candidate gene, expression values reported by eye integration as log_2_(CPM + 1) were obtained for these four tissues, and the maximum tissue-level median value was used as the ocular expression measure. To facilitate comparison across genes, the ocular expression measure was grouped into three empirical score classes: < 1, 1 to < 4.5 and ≥ 4.5 on the log_2_(CPM + 1) scale. The lower boundary of 1 was used to distinguish negligible ocular expression from clearly detectable expression, whereas the upper threshold of 4.5 was chosen as an empirical cut-off to separate moderately from highly expressed genes in the EiaD dataset.

#### Class-specific evidence integration and target scoring

Because Class I and Class II targets were defined under different biological selection frameworks, they were prioritised using class-specific scoring schemes. For Class I common targets, four evidence components were recorded: (i) presence of at least one interaction in the STRING-derived PPI network at a combined score ≥ 0.40; (ii) presence of at least one interaction at a combined score ≥ 0.70; (iii) retinal expression score class derived from the HPA/GTEx datasets; and (iv) ocular expression score class derived from EiaD. Because a STRING combined score ≥ 0.70 indicated stronger interaction confidence than ≥ 0.40, these two PPI indicators were intentionally retained as nested evidence layers, such that targets with high-confidence network support received additional weight beyond basic network connectivity. Higher scores were assigned to higher-confidence PPI categories and higher expression classes, and the component scores were summed to generate a composite score for each Class I target. For Class II myopia-prioritized atropine targets, only transcriptomic evidence was considered, including the retinal expression score class (HPA/GTEx) and ocular expression score class (EiaD). Given the limited size of the Class II set (n = 19), network-based weighting was not applied to avoid overfitting and instability in the topological inference. Therefore, a separate composite score was calculated for Class II targets based on expression evidence alone. Within each class, targets with higher composite scores were considered to have stronger convergent support and were highlighted as priority candidates in the subsequent ranking plots and network visualisations. Composite scores were calculated separately for Class I and Class II targets and used only for within-class prioritization rather than for direct comparison between the two classes.

### GeneMANIA-based functional association network expansion for Class II targets

To explore additional genes functionally related to the 19 Class II myopia-prioritized atropine targets, a functional association network analysis was performed using GeneMANIA (http://www.genemania.org) [34], which integrates multiple genomics and proteomics data types to identify genes with similar functional profiles. For each Class II gene, GeneMANIA was queried under the *Homo sapiens* setting using the default network weighting.

For every query gene, GeneMANIA returns a set of additional genes ranked by the strength of their functional association with the input based on the selected networks. All genes predicted for the 19 Class II targets were pooled, and duplicate entries were removed to obtain a non-redundant set of genes functionally associated with at least one Class II target. The resulting gene list was then merged with the original 19 Class II targets to construct a GeneMANIA-based expanded Class II target set, which served as an extended candidate pool for downstream network visualisation and functional enrichment analysis.

### Retinal single-cell expression analysis

Single-cell RNA- sequencing data for the adult human retina were obtained from a publicly available retinal atlas hosted on the CZ CELLxGENE single-cell portal [35], and cell-type annotations were provided by the accompanying author. The major annotated retinal classes used in this study included retinal ganglion cells (RGCs), bipolar cells, amacrine cells, horizontal cells, rod photoreceptors, cone photoreceptors, Müller glia, astrocytes, microglia, and RPE cells.

For each prioritized Class I or Class II target gene, expression values were extracted from the normalized expression matrix provided on the CZ CELLxGENE portal for all cells belonging to each retinal class. Within every cell-class-gene combination, two summary metrics were computed: (i) the percentage of cells expressing the gene, defined as the proportion of cells with a non-zero expression value in that class, and (ii) the average expression level, calculated as the mean expression across all cells in that class. To facilitate between-gene comparisons within each panel, the average expression values were linearly rescaled to the interval across the corresponding Class I or Class II gene sets and are referred to as scaled expression.

### Molecular docking

To further explore potential interactions between atropine and the prioritized core targets, protein information was first retrieved from the UniProt database, and only entries annotated as "Reviewed" and restricted to *Homo sapiens* were retained*.* The corresponding protein structures were searched for in the RCSB Protein Data Bank (PDB) database (https://www.rcsb.org/) [36]. High-resolution human co-crystal structures of prioritized Class I and Class II Tier 1 targets with bound small-molecule ligands were selected as receptor models for docking (CTNNB1: 7ZRB, MAPK3: 4QTB, AKT1: 4EKL, EGFR: 1M17, HSP90AA1: 3O0I, MAPK1: 4FUY, TP53: 5G4M, CASP3: 1RHJ, PIK3R1: 7PG6, OAT: 7LK0, DUT: 5H4J, BAZ2A: 7BLC, and AKR1B1: 4JIR; see Additional file 2 Table S1). FOS, JUN, and CDC42 proteins were excluded from docking because no suitable human co-crystal structures with small-molecule ligands were available. To ensure structural reliability, receptor structures were selected according to the following criteria: *Homo sapiens* origin, high-resolution experimental structures, near-full-length sequence coverage where possible, and the presence of co-crystallized ligands to define plausible binding regions.

The two-dimensional structure of atropine was obtained from the PubChem database, its three-dimensional conformation was generated, and energy was minimized using Chem3D. For receptor preparation, water molecules, co-crystallized ligands, and irrelevant residues were removed using PyMOL, and the processed protein structures were saved in PDB format. Both the receptor and ligand structures were subsequently prepared in AutoDockTools 1.5.6, and converted into pdbqt format. Molecular docking was performed using AutoDock Vina (https://vina.scripps.edu/) [37, 38]. Additionally, the PROTEINS PLUS web server (https://proteins.plus/) [23] was used to identify the ligand-binding pocket [39]. The docking grid box was centered on the predicted or co-crystallized ligand-binding region, and the pocket box size was set to 22.5 × 22.5 × 22.5 Å for all targets. This box size was selected to fully encompass the ligand-defined binding pocket and adjacent interaction space, while avoiding excessive target-specific tuning. For structural reference, the corresponding co-crystallized ligands were docked into the same ligand-defined binding pocket.

In this study, docking scores were used in a relative manner to compare the binding tendencies of atropine across the core targets. Previous work has suggested that docking scores below approximately − 4.25 kcal/mol generally correspond to acceptable binding, while scores below − 5.0 kcal/mol are often taken to indicate comparatively stronger binding, with values below − 7.0 kcal/mol frequently used as an empirical threshold for high-affinity interactions [40]. To prioritize complexes for subsequent MD simulations while maintaining computational feasibility, only atropine-target complexes with docking scores below − 7.0 kcal/mol were selected as candidate high-scoring models for dynamic evaluation.

### MD simulations

MD simulations were performed using GROMACS (version 2022.2) to investigate the binding interactions of docking complexes [41]. The AMBER ff14SB force field was used for proteins [42], and atropine was parameterized using the General AMBER Force Field with restrained electrostatic potential (RESP) charges [43], which were computed using ORCA 6.0.0 and Multiwfn 3.8 (dev) [44, 45]. The protein–ligand complex for each system was first constructed by combining the individual topology and coordinate files of the protein and ligand, and then centered in an octahedral periodic box with a minimum distance of 1 nm between any protein atom and the box edge to reduce artefactual self-interactions under periodic boundary conditions while keeping the solvent box computationally manageable. The systems were solvated with TIP3P water molecules and neutralized with Na⁺ and Cl⁻ ions.

Energy minimization was performed using the steepest-descent algorithm. The systems were then gradually heated from 0 to 310 K under a constant number of particles, volume, and temperature (NVT) ensemble, followed by 500 ps of sequential NVT and constant number of particles, pressure, and temperature (NPT) equilibration. This equilibration procedure was used to relax unfavourable contacts and stabilize the temperature and pressure before the production runs. A 200 ns production simulation was subsequently conducted in the NPT ensemble under periodic boundary conditions with a 2 fs time step, temperature controlled by the V-rescale thermostat, and pressure maintained by the Parrinello-Rahman barostat [46]. The 200 ns timescale was used as a comparative simulation window to evaluate trajectory stability across complexes. Covalent bonds involving hydrogen atoms were constrained using the LINCS algorithm [47] and long-range electrostatic interactions were treated using the particle-mesh Ewald method. After trajectory correction for the periodic boundary conditions, the root-mean-square deviation (RMSD), root-mean-square fluctuation (RMSF), and radius of gyration (Rg) were calculated. Binding free energies were further estimated using the Molecular Mechanics/Poisson-Boltzmann Surface Area (MM/PBSA) method, based on snapshots extracted from the last 50 ns of each trajectory [48]. The last 50 ns were used because they represented the later, comparatively more stable part of each trajectory for cross-system free-energy estimation. In addition, principal component analysis was performed on the MD trajectories and free-energy landscape maps were generated by projecting conformational ensembles onto the first two principal components (PC1 and PC2) [49].

## Results

### Identification of atropine-, myopia-, and DED-related targets

**Fig. 2:**
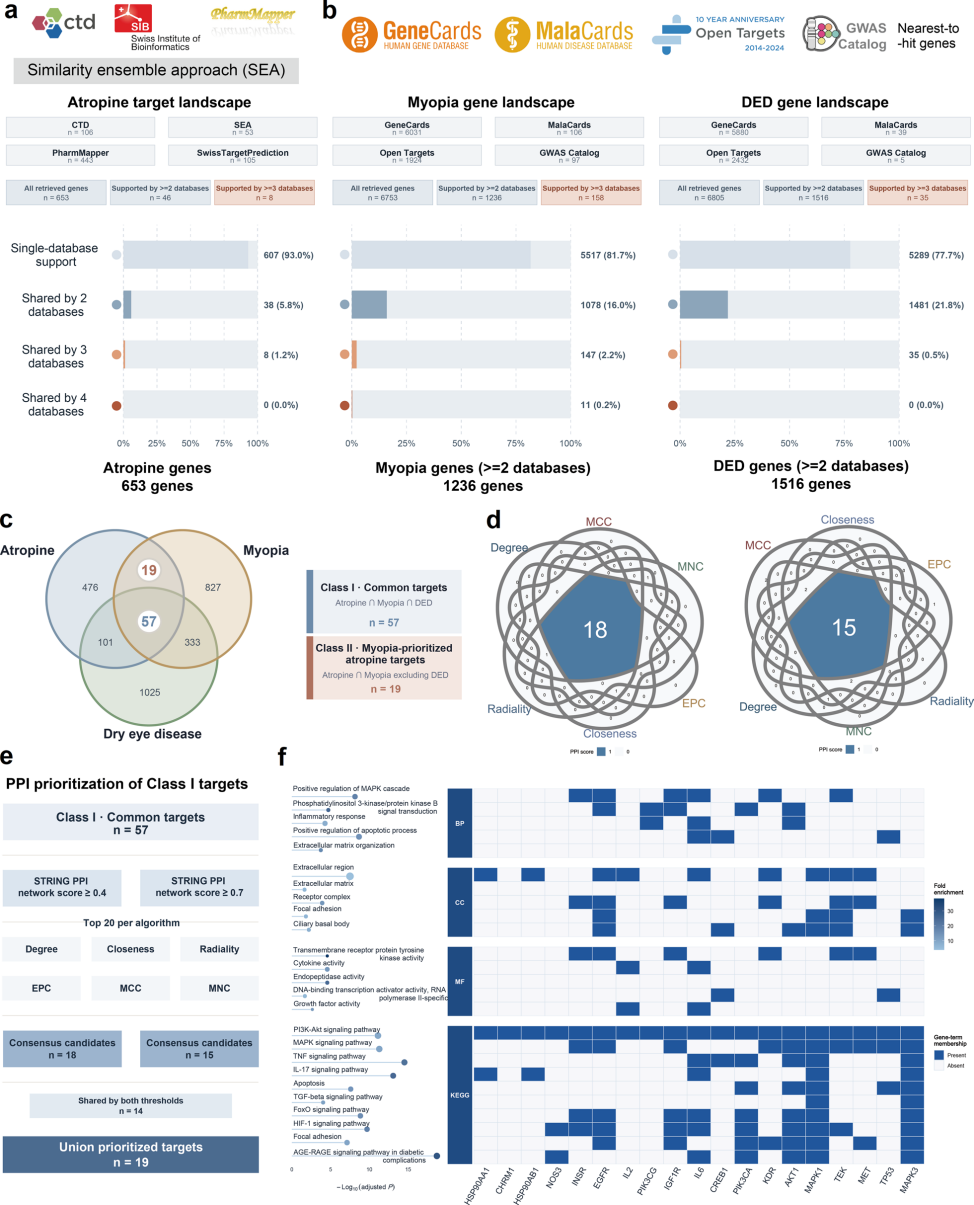
Target collection and Class I network prioritization. **a** Construction of the integrated atropine target landscape from four drug-target resources (SwissTargetPrediction, SEA, CTD, and PharmMapper). Candidate atropine-related proteins were merged across databases, mapped to official human gene symbols, and de-duplicated to obtain the final set of 653 unique targets. **b** Compilation of myopia and dry eye disease (DED) gene landscapes from four disease resources and genome-wide association studies. Disease-associated genes were collected from GeneCards, MalaCards, the Open Targets Platform, and GWAS Catalogue nearest-hit genes, then filtered to retain 1236 myopia genes and 1516 DED genes supported by at least two evidence sources. **c** Venn diagram showing overlap between atropine targets, myopia genes, and DED genes, defining 57 Class I common targets shared by all three sets and 19 Class II myopia-prioritised atropine targets overlapping between atropine and myopia but not DED. **d** STRING-based PPI prioritisation of Class I common targets using six CytoHubba centrality algorithms (MCC, Degree, MNC, EPC, Radiality, and Closeness) under two confidence thresholds (combined score ≥ 0.40 and ≥ 0.70) to identify consensus hub candidates. **e** Schematic overview of the PPI-based prioritisation strategy for Class I targets, integrating consensus hub candidates from both STRING thresholds into a final PPI-prioritised Class I target set for downstream analyses. **f** Functional enrichment profile and gene-term membership pattern of the PPI-prioritized Class I targets, highlighting a shared signalling axis related to inflammation, apoptosis, extracellular matrix remodelling, and growth factor-associated pathways. BP, biological process; CC, cellular component; CTD, Comparative Toxicogenomics Database; EPC, Edge Percolated Component; GO, Gene Ontology; MCC, Maximal Clique Centrality; MF, molecular function; MNC, Maximum Neighbourhood Component; PPI, protein–protein interaction; SEA, Similarity Ensemble Approach; STRING, Search Tool for the Retrieval of Interacting Genes/Proteins

Comprehensive analysis of four public databases identified 653 unique atropine-related targets in humans (Fig. 2a; see Additional file 2 Table S2). Disease-associated genes were compiled from GeneCards, MalaCards, the Open Targets Platform, and genome-wide association studies, resulting in 1,236 myopia-related genes and 1,516 DED-related genes supported by at least two resources (Fig. 2b; see Additional file 2 Table S3). Intersection analysis identified 57 shared targets among atropine, myopia, and DED, which were designated as Class I common targets, and 19 overlapping targets between atropine and myopia that were not associated with DED, which were designated as Class II myopia-prioritised atropine targets (Fig. 2c; see Additional file 2 Tables S4).

### Network prioritization and functional characterization of Class I targets

To prioritize Class I targets at the network level, we evaluated their centrality within the STRING PPI network using two confidence thresholds. At a combined score cut-off of 0.40, 18 genes were consistently ranked within the top 20 by six topological measures (Degree, Closeness, Radiality, EPC, MCC, and MNC) and were therefore retained as consensus PPI candidates. When the STRING threshold was increased to 0.70, 15 genes met the criterion. Fourteen genes overlapped between the two thresholds, and the union of the 0.40- and 0.70-level consensus sets yielded 19 PPI-prioritized Class I targets for subsequent analyses (Fig. 2d–e; see Additional file 2 Table S5).

To functionally contextualize these PPI-prioritized Class I targets, we performed integrated GO and KEGG enrichment analyses. The 57 common Class I genes were significantly enriched for BP related to inflammatory and immune responses, apoptotic signalling, vascular and ECM remodelling, and the regulation of cell proliferation and differentiation (e.g. cellular response to lipopolysaccharide, inflammatory response, and heart and vasculature development). Consistent enrichment signals were also observed at the level of CC (extracellular space/matrix, plasma membrane, and focal adhesion) and MF, such as cytokine activity, growth factor activity, transmembrane receptor protein tyrosine kinase activity, and various kinase/peptidase activities, highlighting their central involvement in extracellular signalling and proteolytic pathways. KEGG pathway analysis further suggested prominent over-representation of PI3K-Akt, MAPK, TNF, IL-17, TGF-β and apoptosis-related pathways, as well as FoxO, HIF-1 and osteoclast-differentiation signalling, underscoring a connected network linking inflammatory cytokine cues with survival, angiogenic and tissue- remodelling cascades (Fig. 2f; see Additional file 2 Table S6). Together, these results suggest that Class I targets converge on a shared inflammatory remodelling signalling axis.

### Ocular transcriptomic prioritisation of Class I targets

**Fig. 3:**
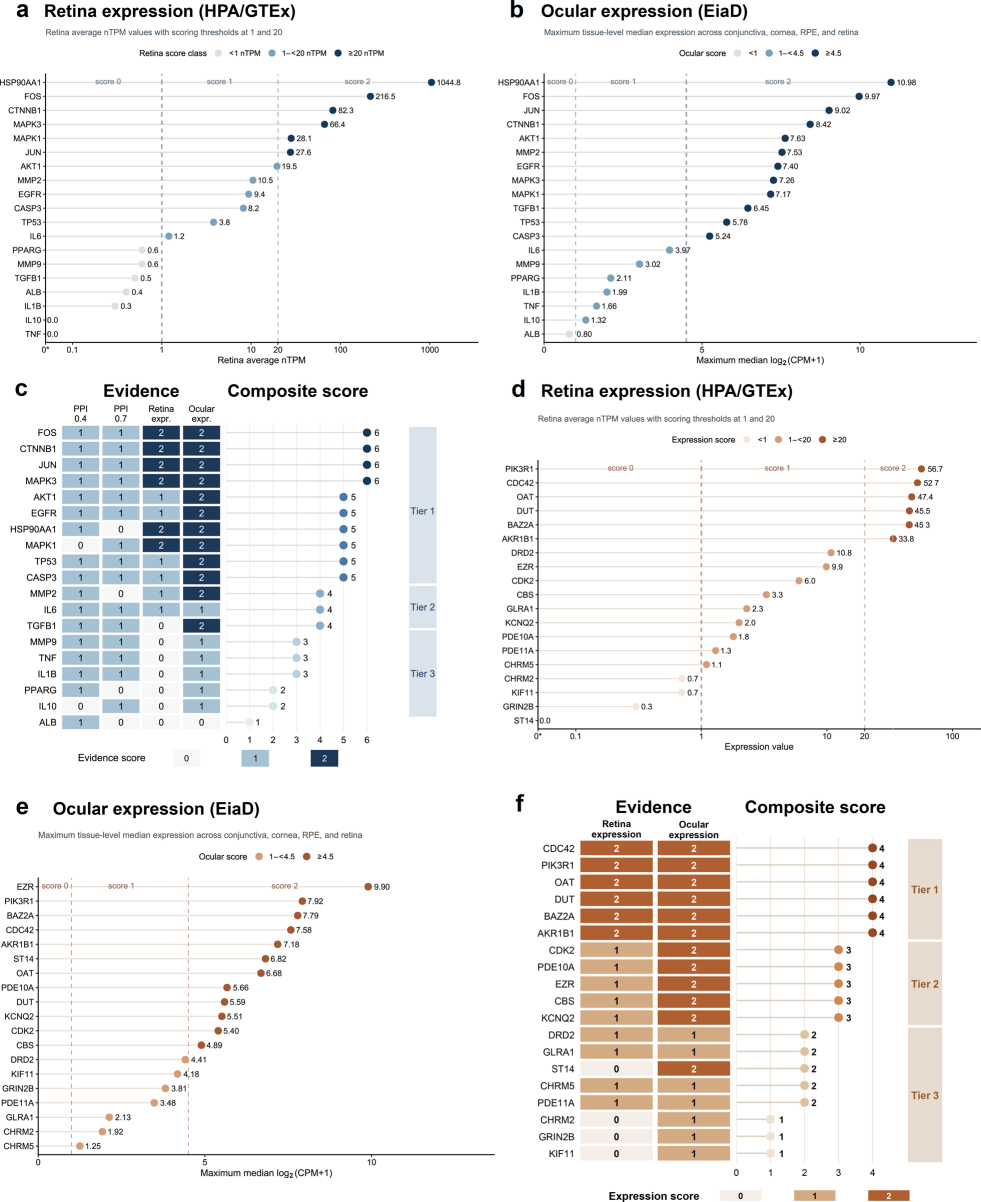
Ocular expression-based prioritisation of Class I and Class II targets. **a** Retina-specific bulk RNA-seq expression of Class I common targets from HPA/GTEx, with genes grouped into three retinal expression-score classes based on nTPM cut-offs (< 1, 1 to < 20, and ≥ 20 nTPM). **b** Pan-ocular bulk RNA-seq expression of Class I common targets from the Eye in a Disk (EiaD/eyeIntegration) resource, summarised as the maximum tissue-level median log_2_(CPM + 1) across conjunctiva, cornea, retina, and retinal pigment epithelium (RPE) and categorised into three ocular expression-score classes. **c** Composite evidence scores and tiering of Class I common targets, integrating STRING PPI support at two confidence thresholds (combined score ≥ 0.40 and ≥ 0.70), retina expression scores, and pan-ocular expression scores to stratify genes into Tier 1–3 priority groups. **d** Retina-specific bulk RNA-seq expression of Class II myopia-prioritised atropine targets from HPA/GTEx, grouped into expression-score classes using the same nTPM thresholds. **e** Pan-ocular bulk RNA-seq expression of Class II myopia-prioritised targets from EiaD/eyeIntegration, summarised and scored as for Class I genes across conjunctiva, cornea, retina, and RPE. **f** Composite expression-based scores and tiering of Class II myopia-prioritised targets, combining retina and pan-ocular expression evidence to stratify the 19 Class II genes into Tier 1–3 candidate groups for downstream functional and structural analyses. CPM, counts per million; EiaD, Eye in a Disk; GTEx, Genotype-Tissue Expression; HPA, Human Protein Atlas; nTPM, normalized transcripts per million; PPI, protein–protein interaction; RNA-seq, RNA-sequencing; RPE, retinal pigment epithelium; STRING, Search Tool for the Retrieval of Interacting Genes/Proteins

To further prioritize the 19 PPI‑derived Class I candidates in an ocular context, retina and eye‑wide expression evidence was integrated with network connectivity into a composite scoring framework. In GTEx/HPA retina bulk RNA‑sequencing data, most candidates indicated at least moderate expression (retina score ≥ 1), with *HSP90AA1*, *FOS*, *CTNNB1*, *MAPK3*, *MAPK1*, *JUN*, and *AKT1* exhibiting particularly high average nTPM values (score 2) **(**Fig. 3a**)**. Evaluation of the conjunctiva, cornea, RPE, and retina in the EiaD atlas was consistent with the robust ocular transcription of these genes, with *HSP90AA1*, *FOS*, *JUN*, *CTNNB1*, *AKT1*, *MMP2*, *EGFR*, *MAPK3*, *MAPK1*, and *TGFB1* ranking among the top expressers at the tissue level **(**Fig. 3b**)**. Summing PPI support (appearance in the 0.40 and/or 0.70 STRING consensus sets), retina expression score and ocular expression score yielded composite evidence scores ranging from 1 to 6, which were used to stratify targets into three tiers: Tier 1 (score 5–6; *FOS*, *CTNNB1*, *JUN*, *MAPK3*, *AKT1*, *EGFR*, *HSP90AA1*, *MAPK1*, *TP53*, *CASP3*), Tier 2 (score 4; *MMP2*, *IL6*, *TGFB1*, *MMP9*) and Tier 3 (score 1–3; *TNF*, *IL1B*, *PPARG*, *IL10*, *ALB*) (Fig. 3c).

### Ocular transcriptomic prioritization of Class II targets

To assess whether pharmacologically tractable Class II targets were transcriptionally supported in the human eye, we first examined bulk retinal expression using GTEx/HPA nTPM values **(**Fig. 3d**)**. Most candidates displayed at least moderate retinal expression (expression score ≥ 1), with *PIK3R1*, *CDC42*, *OAT*, *DUT*, *BAZ2A* and *AKR1B1* reaching high average levels (score 2), whereas *GRIN2B*, *CHRM2*, *KIF11,* and *ST14* indicated only low or negligible expression (score 0–1). Next, we examined tissue-resolved ocular RNA-sequencing data from the EiaD atlas and observed broadly consistent patterns across the conjunctiva, cornea, RPE, and retina, with the same six genes exhibiting strong ocular transcription (ocular score 2) and several additional targets, including *CDK2*, *PDE10A*, *EZR*, *CBS*, and *KCNQ2*, showing intermediate expression (score 1–2; Fig. 3e). Summation of retina and ocular expression scores yielded composite evidence scores ranging from 1 to 4, which were used to stratify the 19 Class II candidates into three tiers: Tier 1 (score 4; *CDC42*, *PIK3R1*, *OAT*, *DUT*, *BAZ2A*, *AKR1B1*), Tier 2 (score 3; *CDK2*, *PDE10A*, *EZR*, *CBS*, *KCNQ2*) and Tier 3 (score 1–2; *DRD2*, *GLRA1*, *ST14*, *CHRM5*, *PDE11A*, *CHRM2*, *GRIN2B*, *KIF11*), thereby highlighting a subset of Class II targets with particularly strong retinal and eye-wide transcriptional support for further consideration (Fig. 3f).

### Functional network expansion of Class II targets

**Fig. 4:**
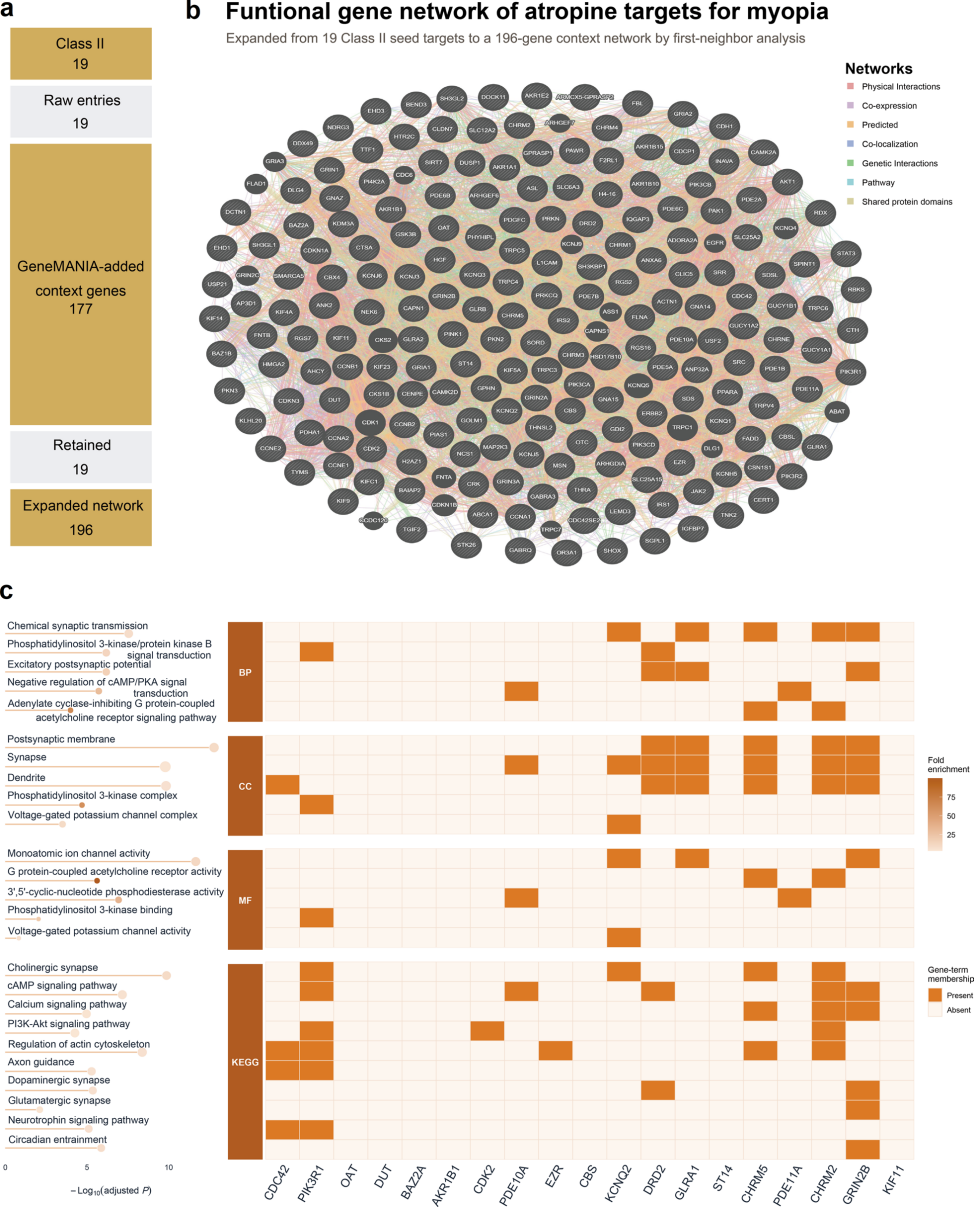
GeneMANIA-based functional association network and enrichment analysis of Class II targets. **a** Workflow for GeneMANIA-based functional association network expansion starting from the 19 pharmacologically tractable Class II myopia-prioritised atropine targets. For each seed gene, first-neighbour genes were retrieved under the *Homo sapiens* setting and pooled across all seeds. **b** GeneMANIA-derived functional association network of the expanded Class II target set. Nodes represent Class II seed genes and GeneMANIA-added context genes; edges reflect functional associations supported by multiple evidence types, including physical interactions, co-expression, predicted interactions, co-localisation, genetic interactions, pathway co-membership, and shared protein domains. **c** Overview of GO and KEGG pathway enrichment results for the 196-gene GeneMANIA-expanded Class II network (19 Class II seeds plus 177 context genes), showing representative significantly enriched terms. **d** Gene-term membership heatmap for prioritised Class II genes across selected enriched GO BP, CC, MF, and KEGG categories, highlighting over-representation of pathways related to synaptic signalling, ion-channel activity, calcium and cAMP signalling, PI3K-Akt signalling, regulation of the actin cytoskeleton, axon guidance, dopaminergic and glutamatergic synapses, and neurotrophin signalling. BP, biological process; cAMP, cyclic adenosine monophosphate; CC, cellular component; GO, Gene Ontology; KEGG, Kyoto Encyclopedia of Genes and Genomes; MF, molecular function

Building on Class II evidence tiers, this study applied GeneMANIA-based functional association network analysis to 19 pharmacologically tractable seed targets. Using first-neighbour expansion, 10 associated genes were retrieved for each seed and merged into a context dataset; after removal of duplicates, this GeneMANIA-expanded set comprised 196 genes in total, including all 19 original Class II targets (Fig. 4a; see Additional file 2 Table S7). The GeneMANIA-expanded set of 196 genes (19 Class II seeds and 177 context genes) constituted a functional gene association network for subsequent enrichment analyses (Fig. 4b).

GO and KEGG enrichment analyses of the 196-gene functional association network identified over-represented terms related to synaptic signalling and ion-channel function (chemical and cholinergic synaptic transmission, voltage-gated potassium channel activity/complex), calcium and cAMP signalling, PI3K-Akt signalling, regulation of actin cytoskeleton and axon guidance, as well as dopaminergic and glutamatergic synapse, and neurotrophin signalling pathways (Fig. 4c; see Additional file 2 Table S8). These enrichments indicate that the broader interaction neighbourhood of Class II targets is preferentially wired into neuronal and second-messenger signalling modules that regulate synaptic activity and cytoskeletal dynamics. This pattern highlights a neuronal signalling layer that is preferentially associated with myopia-specific targets.

### Ocular tissue and retinal single-cell expression patterns of prioritized targets

**Fig. 5:**
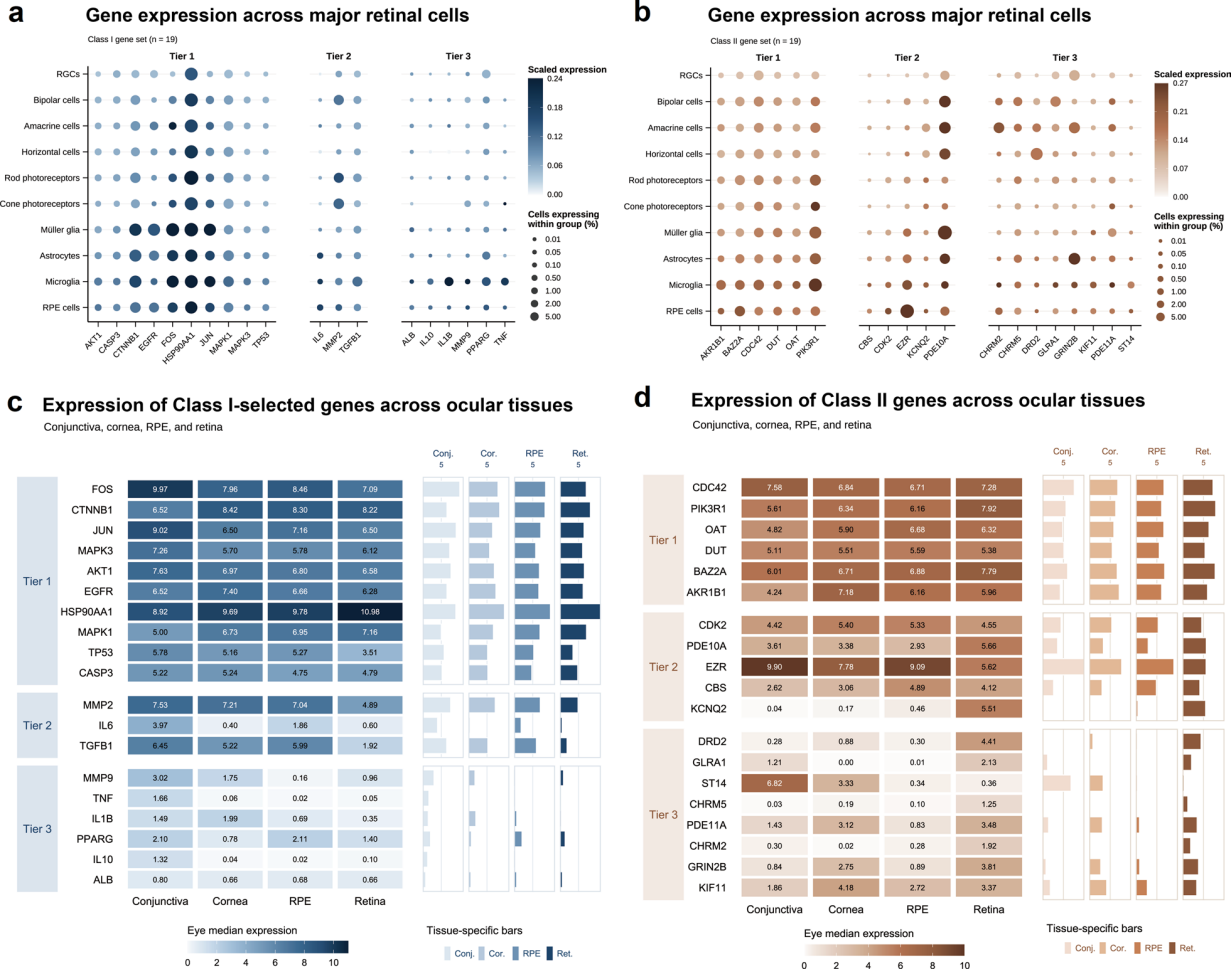
Ocular tissue and retinal single-cell expression patterns of prioritised Class I and Class II targets. **a** Retinal single-cell RNA-seq expression patterns of Class I targets across major retinal cell classes from an adult human retina atlas. Dot size indicates the percentage of cells expressing each gene in a given cell class, and colour intensity reflects scaled average expression; genes are ordered according to the Class I Tier 1–3 prioritisation scheme. **b** Retinal single-cell RNA-seq expression patterns of Class II myopia-prioritised targets across the same retinal cell classes, with dot size representing the fraction of expressing cells and colour intensity indicating scaled average expression, highlighting both broadly expressed and cell type-restricted Class II candidates. **c** Pan-ocular bulk RNA-seq expression landscape of prioritized Class I genes across conjunctiva, cornea, retina, and RPE from the EiaD atlas. Heatmap values represent median log_2_(CPM + 1), and accompanying summaries illustrate tissue-to-tissue differences, showing broadly high expression of Tier 1 genes compared with more limited or tissue-restricted expression of lower-Tier genes. **d** Pan-ocular bulk RNA-seq expression landscape of Class II targets across conjunctiva, cornea, retina, and RPE from EiaD, demonstrating that many Tier 1 Class II genes (*CDC42*, *PIK3R1*, *OAT*, *DUT*, *BAZ2A*, and *AKR1B1*) exhibit consistently strong ocular transcription, whereas several Tier 2–3 candidates show more variable or tissue-biased patterns. CPM, counts per million; EiaD, Eye in a Disk; RGCs, retinal ganglion cells; RPE, retinal pigment epithelium; RNA-seq, RNA-sequencing

To place the candidate targets in a cellular context, we examined their distribution across major retinal cell classes using a public adult human retinal single-cell atlas from the CZ CELLxGENE portal (Fig. 5a–b). For Class I genes, Tier 1 targets such as *FOS*, *CTNNB1*, *JUN*, *MAPK3*, *AKT1*, *EGFR*, and *HSP90AA1* were broadly detected across neuronal populations, with particularly high scaled expression and cell-level prevalence in bipolar, amacrine, and Müller glial cells, whereas Tier 3 genes (*TNF*, *IL1B*, *PPARG*, *IL10*, and *ALB*) showed generally lower expression and were restricted to smaller fractions of microglia, astrocytes, and RPE cells (Fig. 5a). Class II genes displayed a complementary pattern: Tier 1 targets (*AKR1B1*, *BAZ2A*, *CDC42*, *DUT*, *OAT*, and *PIK3R1*) were enriched in multiple interneuron classes and Müller glia, whereas several lower-Tier genes, including ion- channels and neurotransmission-related targets such as *KCNQ2*, *DRD2*, and *GRIN2B*, showed more selective expression in photoreceptors, bipolar cells, and microglia (Fig. 5b).

Next, we assessed the bulk tissue expression of the same targets across key ocular tissues (conjunctiva, cornea, RPE, and retina) using the Eye Integration Atlas (EiaD; Fig. 5c–d). Among Class I genes, *FOS*, *CTNNB1*, *JUN*, *MAPK3*, *AKT1*, *EGFR*, *HSP90AA1*, and *MAPK1* exhibited consistently high median expression across all four tissues, with *HSP90AA1* showing the strongest signal in the retina, whereas Tier 3 genes, such as *TNF*, *IL1B*, *PPARG*, *IL10*, and *ALB*, displayed modest or tissue-specific expression (Fig. 5c). For Class II genes, Tier 1 targets (*CDC42*, *PIK3R1*, *OAT*, *DUT*, *BAZ2A*, and *AKR1B1*) showed uniformly high expression across ocular tissues, while several Tier 2 and Tier 3 candidates (e.g. *EZR*, *KCNQ2*, *DRD2*, *GLRA1*, *GRIN2B*, and *KIF11*) exhibited more variable, tissue-biased patterns, with *EZR* and *KCNQ2* relatively enriched in the cornea and retina, and *GRIN2B* showing low but detectable expression primarily in the retina (Fig. 5d).

### Molecular docking analysis of prioritised Class I and Class II targets

**Fig. 6:**
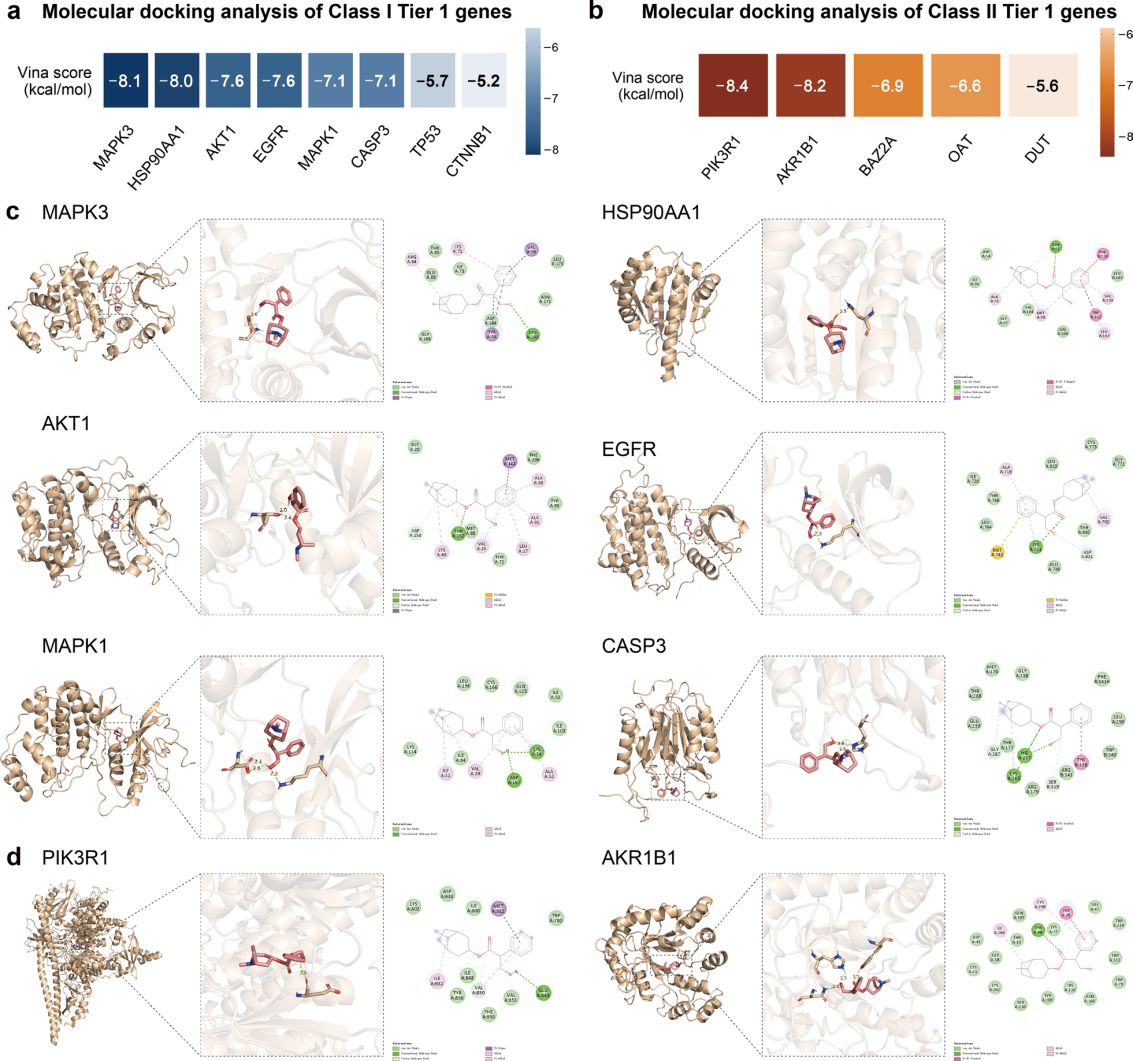
Molecular docking analysis of prioritized Class I and Class II Tier 1 targets. **a** AutoDock Vina docking scores for atropine with structurally eligible Tier 1 Class I targets (MAPK3, HSP90AA1, AKT1, EGFR, MAPK1, CASP3, TP53, and CTNNB1), illustrating that several kinases and chaperone proteins show relatively favourable predicted binding (around or below − 7.0 kcal/mol). **b** Docking scores for atropine with structurally eligible Tier 1 Class II targets (PIK3R1, AKR1B1, BAZ2A, OAT, DUT), indicating comparatively stronger predicted binding for PIK3R1, AKR1B1, BAZ2A, and OAT than for DUT. **c** Representative 3D binding poses and 2D interaction maps for selected Class I complexes (MAPK3, AKT1, MAPK1, HSP90AA1, EGFR, and CASP3), showing that atropine occupies canonical or co-crystallised ligand pockets and forms one or more conventional hydrogen bonds with key active-site residues, together with extensive hydrophobic and π-related interactions. **d** Representative 3D binding poses and 2D interaction maps for selected Class II complexes (PIK3R1, AKR1B1, and OAT), highlighting hydrogen bonds with residues such as GLU849 (PIK3R1), ASN51 (AKR1B1), and HIS237 (OAT), as well as complementary van der Waals contacts that support a well-packed binding mode. Vina, AutoDock Vina docking score; MD, molecular dynamics

Molecular docking was used to assess the propensity of atropine to bind to Class I and Class II Tier 1 targets (Fig. 6a–b). For structural comparison, the corresponding co-crystallised reference ligands were docked in the same ligand-defined pockets, and the comparative docking scores are summarized in Additional file 2 Table S9. Among Class I Tier 1 proteins, MAPK3, HSP90AA1, AKT1, EGFR, MAPK1, and CASP3 all showed favourable predicted binding, with Vina scores ranging from − 7.1 to − 8.1 kcal/mol, whereas TP53 and CTNNB1 had more modest scores of − 5.7 and − 5.2 kcal/mol, respectively (Fig. 6a). Detailed inspection of the best-scoring poses suggested that atropine established one or more conventional hydrogen bonds with key active-site residues in MAPK3 (ASP184), AKT1 (THR152), MAPK1 (ASP167 and LYS54) and CASP3 (HIS110, ASN160 and TYR48), complemented by multiple hydrophobic and π-related contacts that helped anchor the ligand within the canonical binding clefts **(**Fig. 6c**)**.

For Class II Tier 1 targets, atropine exhibited similarly favourable docking scores towards PIK3R1, AKR1B1, BAZ2A and OAT (− 8.4 to − 6.6 kcal/mol), while DUT showed a weaker predicted interaction (− 5.6 kcal/mol) (Fig. 6b). In the top-ranked complexes, atropine formed stable hydrogen bonds with the residues located in the functional pockets of PIK3R1 (GLU849), AKR1B1 (ASN51), and OAT (HIS237), together with extensive van der Waals and hydrophobic contacts around the bicyclic core, indicating a well-packed binding mode for these targets (Fig. 6d).

### MD simulations of prioritized atropine-target complexes

**Fig. 7:**
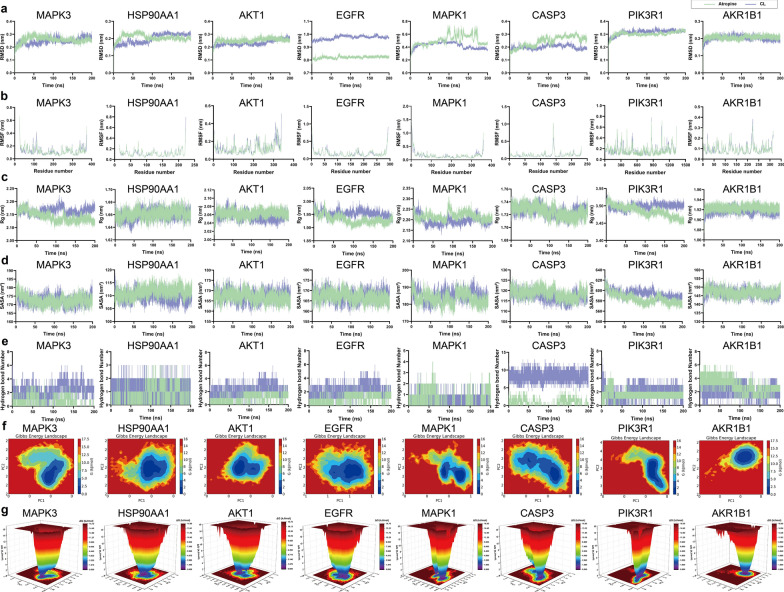
MD simulations of atropine-bound and co-crystallized ligand (CL)-bound complexes. **a** Time evolution of backbone RMSD during 200 ns MD simulations for eight selected protein–ligand systems (MAPK3, HSP90AA1, AKT1, EGFR, MAPK1, CASP3, PIK3R1, and AKR1B1), comparing atropine-bound complexes with the corresponding CL-bound reference structures. **b** Per-residue RMSF profiles for the same systems, showing largely overlapping flexibility patterns between atropine-bound and CL-bound states, with elevated fluctuations mainly confined to loop and terminal regions. **c** Time evolution of the radius of gyration (Rg) for each complex, indicating that global compactness is generally maintained throughout the simulations in both atropine-bound and CL-bound conditions. **d** Time evolution of SASA, demonstrating the absence of systematic increases in SASA for atropine-bound complexes relative to CL-bound references, consistent with preserved overall structural integrity. **e** Time-resolved hydrogen-bond analysis between ligand and protein for each system, revealing target-dependent differences in hydrogen-bond occupancy but continuous polar contacts for atropine in all complexes. **f** Two-dimensional FELs derived from principal component analysis (PC1 and PC2) of the MD trajectories for atropine-bound complexes, showing that most systems occupy one or a few well-defined low-energy basins. **g** Three-dimensional representation of the corresponding Gibbs free-energy landscapes for atropine-bound complexes, illustrating the depth and distribution of dominant conformational minima and supporting the dynamic stability of the simulated binding modes. MD, molecular dynamics; PC1, principal component 1; PC2, principal component 2; RMSD, root-mean-square deviation; RMSF, root-mean-square fluctuation; SASA, solvent-accessible surface area

To further evaluate the structural compatibility of atropine with the prioritized targets, 200 ns MD simulations were performed for eight Class I/Class II Tier 1 complexes (MAPK3, HSP90AA1, AKT1, EGFR, MAPK1, CASP3, PIK3R1, and AKR1B1), and each atropine-bound system was compared with the corresponding co-crystallized ligand-bound reference state (Fig. 7a–g). Overall, the atropine-bound trajectories reached stable plateaus during the early phase of the simulations and subsequently remained within relatively restricted fluctuation ranges, indicating that all eight binding pockets accommodated atropine without overt structural destabilisation (Fig. 7a). The RMSF, Rg, and solvent-accessible surface area (SASA) profiles were generally comparable between the atropine- and co-crystallized ligand-bound states, with most differences confined to flexible peripheral regions (Fig. 7b–d). Among the eight systems, EGFR showed one of the more regular dynamic profiles, with comparatively smooth RMSD, Rg, and SASA trajectories; MAPK1 exhibited greater mid-trajectory variability; and HSP90AA1, AKT1, and CASP3 showed somewhat broader oscillations (Fig. 7a–d).

Hydrogen bond analysis revealed target-dependent interaction behaviour (Fig. 7e). In HSP90AA1, MAPK1, and AKR1B1, atropine maintained hydrogen bond occupancy comparable to or slightly higher than that of the corresponding co-crystallized ligand-bound states, whereas CASP3 and PIK3R1 exhibited more persistent hydrogen bonding in the reference ligand-bound systems. Consistent with these observations, free-energy landscape analysis showed relatively concentrated low-energy basins for several complexes, including EGFR, whereas MAPK1 and PIK3R1 sampled a broader conformational space (Fig. 7f–g). MM/PBSA analysis further refined the energetic ranking (see Additional file 2 Table S10): the most favourable binding free energies were observed for AKT1 (ΔGbind =  − 29.68 ± 4.34 kcal/mol), AKR1B1 (− 26.42 ± 4.70 kcal/mol), and MAPK1 (− 23.46 ± 4.96 kcal/mol), followed by PIK3R1 (− 19.52 ± 3.90 kcal/mol) and EGFR (− 19.28 ± 5.83 kcal/mol), whereas HSP90AA1 (− 15.70 ± 4.70 kcal/mol), MAPK3 (− 11.69 ± 5.87 kcal/mol), and CASP3 (− 7.29 ± 6.27 kcal/mol) were less favourable. Taken together, these results indicate that AKT1, AKR1B1, and MAPK1 are the most favourable atropine-compatible complexes in energetic terms, whereas EGFR emerges as a structurally well-accommodated target with a comparatively stable dynamic behaviour.

## Discussion

This study used an integrative in silico framework to characterize the potential target landscape of atropine in myopia and DED. By combining cross-database target mining, network analysis, ocular transcriptomic prioritization, molecular docking, and MD simulations, this study identified two related but distinguishable target layers: a shared Class I layer enriched in inflammatory, ECM remodelling, apoptotic, and growth factor-associated pathways, and a myopia-prioritized Class II layer linked more strongly to neuronal, ion-channel, cytoskeletal, and second-messenger signalling. Rather than supporting a single canonical mechanism, these findings suggested that atropine-related ocular biology is organised across multiple interconnected pathways.

Atropine remains the most widely used pharmacological intervention for the control of myopia; however, its mechanism of action is not completely understood [50]. Current evidence suggests that its anti-myopia effects cannot be explained solely by cycloplegia or accommodation blockade but are more likely to involve receptor- and tissue-level actions across the retina, RPE, choroid, and sclera, including muscarinic and possibly non-muscarinic signalling pathways [3, 51]. Experimental data have also implicated muscarinic receptor subtypes in myopia susceptibility, supporting the idea that atropine acts within a broader signalling network [52, 53]. Against this background, our findings support the view that atropine-related biology in myopia and DED is likely to be organized as a multilayered target network, rather than a single canonical pathway.

One of the major findings of the present study is that the shared Class I target set converged on inflammatory, apoptotic, ECM and growth-factor-related signalling pathways, including MAPK, PI3K-Akt, TGF-β and apoptosis-associated modules [54, 55]. This pattern is biologically meaningful because accumulating evidence indicates that myopia progression is not purely biomechanical but is closely linked to inflammatory and remodelling processes, including IL-6-, TGF-β- and MMP-associated pathways involved in scleral ECM turnover and axial elongation [55–57]. Recent work has further connected myopia-related phenotypes to PI3K-Akt/NF-κB signalling and downstream changes in *TGFB1*, *MMP2* and *IL6* in retinal and scleral tissues [58], supporting the concept of a "common stress/remodelling axis" in ocular growth regulation.

From a dry-eye perspective, the same Class I axis aligns with established inflammatory and epithelial-repair programmes on the ocular surface. Ocular surface disease is strongly associated with inflammatory cytokines, MAPK activation and matrix- remodelling enzymes such as MMP-9, and classic experimental work has shown that experimental dry eye stimulates IL-1β, TNF-α, MMP-9 and MAPK signalling in corneal and conjunctival epithelia [59, 60]. MMP-9 has since become one of the most widely used biomarker-linked pathways for DED severity, while EGFR signalling is well recognized as a key regulator of corneal epithelial maintenance and wound healing, further supporting the ocular plausibility of retaining EGFR-related signalling within this shared network rather than dismissing it as purely "cancer-related" [59, 61]. Accordingly, the Class I targets in our network were best interpreted as candidate mediators of overlapping inflammatory, epithelial-repair, and matrix- remodelling programmes that could intersect with both myopia biology and ocular-surface vulnerability. In this framework, Class I does not imply that atropine directly causes DED through these targets but rather highlights a shared stress-response interface through which beneficial modulation of scleral remodelling and context-dependent ocular-surface effects may partially overlap.

In contrast, Class II myopia-prioritised targets and their GeneMANIA-expanded network showed a much stronger relationship with synaptic signalling, ion-channel activity, calcium/cAMP signalling, actin-cytoskeleton regulation, and neurotrophin-associated pathways. This distinction is important because contemporary models of myopia increasingly place retinal neurotransmission and downstream retinal-to-scleral signalling at the center of ocular growth regulation rather than viewing myopia control solely through ciliary muscle accommodation [62]. Classical "muscarinic" hypotheses emphasise M-receptor blockade and cholinergic pathways, but recent reviews and experimental antagonist studies indicate that atropine's anti-myopia effects also intersect dopaminergic, second-messenger and other neuronal signalling modules [3, 53, 63]. In this context, our Class II network may capture a more myopia-centred neuronal signalling layer that operates alongside, and highlights additional intracellular and neurosignaling candidates with ocular transcriptional support that warrant follow-up in mechanistic and translational studies.

Therefore, the ocular surface effects of atropine should be interpreted with caution. Clinical evidence does not support the simple conclusion that low-dose atropine uniformly induces DED, and prospective data with 0.01% atropine did not show sustained deterioration in tear quantity or stability over several months of use. More recent work suggests that short-term topical atropine can transiently reduce tear meniscus height and increase dry- eye-related symptoms, with more noticeable but reversible effects at higher concentrations such as 0.05% [9, 64]. Our findings do not support the conclusion that atropine directly causes DED through prioritized Class I targets. Instead, the shared Class I network may represent a biological interface at which pathways relevant to scleral remodelling, inflammatory signalling, epithelial repair, and ocular surface stress partially intersect. Within this framework, atropine-related dry eye symptoms are more plausibly viewed as context-, concentration-, and susceptibility-dependent phenomena than as inevitable pharmacological outcomes.

Collectively, our findings support a two-layered view of atropine-related biology of the eye. First, a shared Class I axis centered on inflammatory, ECM‑remodelling, apoptotic and growth‑factor signalling appears to link scleral remodelling in myopia control with epithelial‑repair and stress responses on the ocular surface, but does not on its own prove that atropine directly causes DED. Second, a myopia‑prioritized Class II axis enriched for synaptic, ion‑channel, PI3K-Akt and neurotrophin pathways points to a more retinal‑centered neuronal signalling layer that may help explain heterogeneous treatment responses and suggests candidate targets for adjunct or alternative myopia therapies beyond canonical muscarinic receptors. Viewed in this way, proteins such as AKT1, MAPK1/3, EGFR, HSP90AA1, PIK3R1 and AKR1B1 are best considered biologically supported nodes within these two axes that merit focused experimental validation and prospective clinico‑transcriptomic studies.

From a clinical perspective, the shared Class I axis may, therefore, be more informative as an efficacy-tolerability interface than as a direct causal pathway. Many of its Tier 1 nodes, such as *EGFR*,* MAPK1/3*, *AKT1*, *HSP90AA1*, *MMP2/9*, and *TGFB1*, sit at the crossroads of scleral remodelling, inflammatory signalling, and epithelial-repair programmes, suggesting that inter-individual variation along this axis could modulate both myopia control efficacy and the propensity for ocular-surface symptoms under topical atropine. In future, Class I signatures or downstream readouts (for example, tear‑film MMP‑9 or EGFR‑related epithelial markers) might be explored as biomarkers to identify children who require closer ocular‑surface monitoring or adjunctive anti‑inflammatory or tear‑stabilizing therapy when receiving atropine. By contrast, the myopia-prioritized Class II axis has direct implications for understanding and personalizing myopia control. Its enrichment for synaptic, ion‑channel, PI3K-Akt and neurotrophin pathways, together with strong retinal and ocular expression of targets such as *CDC42*, *PIK3R1*, *OAT*, *DUT*, *BAZ2A* and *AKR1B1*, suggests that variability along this neuronal layer could contribute to heterogeneous atropine responsiveness between patients. In the longer term, Class II signatures may help to (i) stratify children by likelihood of benefiting from low‑dose atropine and (ii) nominate adjunct or alternative targets that modulate retinal signalling to enhance myopia control while potentially permitting lower muscarinic blockade and fewer ocular‑surface side‑effects.

Our structural analyses provide further support for this prioritization. Docking and MD results suggested that several Tier 1 Class I and Class II proteins could accommodate atropine within plausible ligand-binding regions, and that a subset of atropine-bound complexes remained dynamically stable relative to their co-crystallized ligand-bound reference states. This study interprets these structural findings as supportive, comparative evidence that helps rank candidate atropine-target interactions for future validation.

This study had certain limitations. Although the study reduced database-specific noise by cross-resource filtering and added retinal, pan-ocular, and retinal single-cell expression support, the study still lacked matched transcriptomic information for some highly relevant tissues such as the sclera, meibomian gland, and lacrimal gland. In addition, the literature‑supported plausibility of pathways such as MAPK, PI3K-Akt, TGF‑β, MMP and EGFR in ocular biology does not prove atropine‑dependent regulation of those pathways in vivo. Finally, our docking and MD simulation workflow does not replace the need for biochemical binding assays, perturbation experiments, or pharmacokinetic confirmation of target accessibility. Therefore, this study should be viewed as a hypothesis-generating, biologically contextualized prioritization framework.

## Conclusions

This study identified a shared Class I target layer that potentially links atropine-related biology across myopia and DED, together with a myopia-prioritized Class II target layer that may be more relevant to retinal neuronal signalling and myopia control. By integrating multi-resource target mining, network and enrichment analyses, ocular transcriptomic prioritisation, molecular docking, and comparative MD evaluation, this study provides a biologically contextualised framework for prioritizing candidate atropine-associated targets and pathways.

## Supplementary Information

Additional file 1 (DOCX 17 KB)

Additional file 2 (XLSX 502 KB)

## Data Availability

No datasets were generated or analysed during the current study.

## Abbreviations

DED: Dry eye disease
GO: Gene ontology
KEGG: Kyoto encyclopedia of genes and genomes
PPI: Protein–protein interaction
MD: Molecular dynamics
MM/PBSA: Molecular mechanics/poisson-boltzmann surface area
ECM: Extracellular matrix
VIP: Vasoactive intestinal peptide
SMILES: Simplified molecular input line entry system
SEA: Similarity ensemble approach
CTD: Comparative toxicogenomics database
GWAS: Genome-wide association study
LD: Linkage disequilibrium
BP: Biological process
CC: Cellular component
MF: Molecular function
MCC: Maximal Clique Centrality
MNC: Maximum Neighbourhood Component
EPC: Edge percolated component
HPA: Human Protein Atlas
GTEx: Genotype-Tissue Expression
nTPM: Normalized transcripts per million
EiaD: Eye in a Disk
RPE: Retinal pigment epithelium
CPM: Counts per million
RGCs: Retinal ganglion cells
PDB: Protein Data Bank
RESP: Restrained electrostatic potential
RMSD: Root-mean-square deviation
RMSF: Root-mean-square fluctuation
Rg: Radius of gyration
PC1: Principal component 1
PC2: Principal component 2
SASA: Solvent-accessible surface area
