# Supplementary material for: Network pharmacology and molecular dynamics simulations reveal shared mechanisms and myopia-specific targets of atropine in myopia and dry eye disease
Source: Eye Vis (Lond). 2026 Jun 22;13:27. doi: 10.1186/s40662-026-00493-1 (PMC13285102; doi:10.1186/s40662-026-00493-1)
Supplement: Supplementary file 1 — Additional file 1 (DOCX 17 KB) [file 40662_2026_493_MOESM1_ESM.docx]

**Additional file 1**

**Additional file 1a. Detailed retrieval and harmonization of atropine-related targets**

In SwissTargetPrediction, Similarity Ensemble Approach (SEA), and PharmMapper, all returned entries annotated as *Homo sapiens* were retained during the initial collection stage. In the Comparative Toxicogenomics Database (CTD), curated human atropine-gene interaction records were extracted, and nonhuman or nonprotein-coding entries were excluded where applicable. After retrieval, all candidate targets were pooled into a single list. Protein names and gene identifiers were harmonized with official human gene symbols using UniProt, and duplicate entries arising from overlapping database coverage, synonym usage, or repeated records were removed after symbol standardization.

**Additional file 1b. Detailed retrieval and filtering of disease-associated genes for myopia and dry eye disease (DED)**

For GeneCards, MalaCards, and the Open Targets Platform, the search terms "myopia" and "dry eye disease" or "dry eye syndrome" were used. The returned gene lists were exported and combined after symbol harmonization. For the Genome-Wide Association Studies (GWAS) layer, summary statistics from GCST90475880 and GCST90475895 were processed by selecting variants that reached genome-wide significance (*P* < 5 × 10^−8^), followed by linkage disequilibrium (LD) clumping in PLINK version 1.9, with 1000 genome reference genotypes matched to the harmonized genome build. Within each LD clump, the lead single-nucleotide polymorphism (SNP) was defined as the variant with the smallest *P-*value, and the nearest protein-coding gene was assigned as the nearest-to-hit gene. After harmonization across all resources, duplicate genes were removed, and support counts were tabulated for downstream evidence-based filtering.
